# Persistent post-concussive syndrome in children after mild traumatic brain injury is prevalent and vastly underdiagnosed

**DOI:** 10.1038/s41598-022-08302-0

**Published:** 2022-03-14

**Authors:** Eli Fried, Uri Balla, Merav Catalogna, Eran Kozer, Adi Oren-Amit, Amir Hadanny, Shai Efrati

**Affiliations:** 1 Pediatric Division, Kaplan Medical Center, Rehovot, Israel; 2 The Sagol Center for Hyperbaric Medicine and Research, Shamir (Assaf Harofeh) Medical Center, 70300 Zerifin, Israel; 3 Pediatric Division, Shamir Medical Center, Zerifin, Israel; 4 Sackler School of Medicine and the Sagol School of Neuroscience, Tel-Aviv University, Tel Aviv, Israel

**Keywords:** Neurological disorders, Trauma, Paediatric research

## Abstract

Data on epidemiology and prognosticators of persistent post-concussion syndrome (PPCS) after mild traumatic brain injury (mTBI) in the pediatric population is scarce. The aim of this study was to evaluate the prevalence of PPCS in children after mTBI and to identify clinical variables in children who are at high risk for developing PPCS. A multicenter, retrospective matched cohort in which PPCS symptoms were evaluated in children 8–15-year-old, 6–60 months after being admitted to the emergency department because of mTBI. The control group included children admitted to the emergency department because of uncomplicated distal radius fractures. The children's guardians were interviewed for the presence of PPCS symptoms using the "Rivermead Post-Concussion Questionnaire". A multivariable logistic regression model was used to identify predictors of PPCS. Two-hundred and five children were included in the mTBI group and 205 in the control. The median time from the injury was 33.5 months in the mTBI group and 33.8 in the control. The prevalence of PPCS in the mTBI group was 25.3% and PPCS like symptoms in the control was 2.4%, *p* < 0.001. Within the 6–60 months period, the PPCS prevalence was not influenced by the time that elapsed from the injury. In the mTBI group, motor vehicle accidents and adolescence were found to be risk factors for PPCS. PPCS is underdiagnosed in the pediatric population and 25% of children admitted to the ED due to mTBI may suffer from PPCS. Screening guidelines should be implemented to identify and properly treat these children.

## Introduction

Traumatic brain injury (TBI) is one of the most common reasons for emergency department (ED) visits with a reported 150–400 visits per 100,000 children^1–3^. Even though most of these are considered mild traumatic brain injury (mTBI)^4^, with normal brain imaging, many will suffer from post-concussion syndrome (PCS) that consists of somatic, cognitive and emotional symptoms^5–7^. In the weeks following the mTBI, PCS symptoms are expected to resolve in most patients. However, in some cases, PCS does not resolve, and the symptoms become chronic. This is referred to as persistent post-concussion symptoms (PPCS)^8^.

The prevalence of PPCS among children after mTBI is not well known, with reports ranging from 2.3 to 33%^9–11^. Most studies done on pediatric populations focused on short to intermediate intervals, weeks to months after the injury. We found only three studies that evaluated the prevalence of PPCS a year after the acute trauma^12–14^. One study, conducted by Barlow et al. evaluated the prevalence of PPCS symptoms in children after mTBI compared to children who came to the ED with extracranial injury (ECI)^12^. They found that the prevalence of persistent symptoms at 1 year was 2.3% in the mTBI group and 0.01% in the ECI group^12^. In another study, recently done by Ewing-Cobbs et al. it was found that one year after mTBI, 25% of children suffered from PPCS^13^.

The aim of this study was to evaluate the prevalence of PPCS symptoms in children, 6–60 months after mild TBI. The prevalence was compared to a control group that included a matched population of children who visited the ED with ECI and suffered from a fracture of the distal radius. The secondary objective of this study was to identify potential clinical variables that can mark the children who are at high risk for developing PPCS.

## Methods

A multicenter, retrospective matched cohort in which the prevalence of persistent PCS symptoms was evaluated in children who visited the ED due to mTBI or due to uncomplicated distal radius fracture between 2015 and 2020. The research was conducted in two Israeli hospitals, and was approved by Shamir Medical Center’s, and Kaplan Medical Center’s institutional review boards (IRB) (No. 029-21-ASF, 0179-20-KMC). The study was conducted in accordance with The World Medical Association Declaration of Helsinki. Verbal informed consent from the patients' parents was obtained. Verbal informed consent has been deemed sufficient for inclusion in this research by both Shamir Medical Center’s and Kaplan Medical Center’s IRB.

### Study population

The hospital electronic databases were used to screen for children aged 8–15 years, who their injury occurred 6–60 months prior to their inclusion. Children were included in the mTBI group if they met the criteria defined by the "Mild Traumatic Brain Injury Committee of the American Congress of Rehabilitation Medicine". They define mTBI as an event in which the head has been struck or if the brain had an acceleration/deceleration movement after which the Glasgow Coma Scale (GCS) score was 13–15, loss of consciousness (LOC) or an altered mental state lasted less than 30 min, an absence of focal neurologic deficits, and posttraumatic amnesia of no more than 24 h^15,16^.

The control group included children with extracranial injuries (ECI) of similar age and time elapsed from injury, who visited the EDs of these hospitals because of uncomplicated distal radius fractures. The control group patients were randomly selected by matching age, sex, and duration from the insult with the mTBI group.

Children were excluded if they had preexisting neurological, neurosurgical, or psychiatric problems. Children were also excluded if they were hospitalized for more than 48 h, suffered multi-trauma injuries, or if the computed tomography (CT) scan showed evidence of traumatic intracranial findings.

The hospital electronic databases were used to identify children who were hospitalized or had a head CT scan due to their injury and received the diagnosis in their medical record of "head injury", "fall accident", "concussion" or "motor vehicle accident". For the ECI group, the relevant diagnoses were "torus fracture of radius (alone), "fracture of the radius and ulna", "fracture of radius" or "fracture of upper limb". These identified cases were then reviewed by our research team for eligibility for inclusion as detailed above.

### Data collection

Parents of patients who were eligible for inclusion in the study were contacted by telephone, and voluntary verbal informed consent was obtained from the patients' parents. PPCS symptoms were assessed using the Rivermead Post-Concussion Questionnaire (RPQ), which is a 16-item symptom inventory checklist, which has been used in pediatric studies of mTBI^17–20^. The questionnaire was filled solely by the patients' parents as an online form or by a telephone interview.

Medical records were reviewed for clinically significant information, including demographic details, mechanism of TBI, signs and symptoms of TBI, physical examination on admission and CT scan results (if performed). The mechanism was categorized as: fall accident, sports related, motor vehicle accident (MVA) or other (assault, direct blunt trauma, etc.). The demographic variables included: age at the time of the injury, sex, and time since the incident. The clinical variables collected included LOC, vomiting, headache, mental status changes (i.e., restlessness, somnolence, or confusion), contusion signs seen upon physical examination and CT scan results. Age was categorized into two subcategories: school age (< 13 years) and adolescent (13 years and up).

We defined PPCS as the presence of three or more symptoms on the RPQ that were worse than before the injury. This approach is in accordance with the diagnostic criteria for PCS as defined by the tenth edition of the "International Classification of Diseases" (ICD 10)^7^.

PPCS positive questionnaires were further analyzed for symptom groups, as has been done in past studies^21,22^. The three symptom categories are: cognitive (forgetfulness, poor concentration, taking longer to think), somatic (headaches, double or blurred vision, sensitivity to noise, dizziness, nausea, sleep disturbance, fatigue) and emotional (irritability, depression, frustration, restlessness).

### Statistical analysis

Descriptive statistics: Continuous data are expressed as means ± standard-deviations (SD), and as median and interquartile range (IQR). Independent t-tests with a two-tail distribution were performed to compare variables between groups, when a normality assumption held according to a Kolmogorov–Smirnov test. Categorical data were expressed in numbers and percentages and compared by using chi-square tests or Fisher’s exact tests. The Mann–Whitney U test was used to compare between RPQ scores. Chi-square test of independence 5 × 2 model was used to test independence of post-injury presence of PPCS across years. A value of *p* < 0.05 was considered significant.

Adjusted odds ratios (OR) and the 95% confidence intervals (95% CI) were calculated using univariate and multivariable logistic regression models to identify significant predictors of PPCS. Model variables included demographics, mechanism of injury, and clinical factors observed during the ED admission. Validity of the multivariate logistic regression model was further assessed by performing bootstrap validation resampling technique with 1000 samples^23^.

Sample size estimation: Based on previous studies, the prevalence of PPCS among children ranges from 2.3 to 33%^9–11^, while the incidence of PPCS in distal radius fracture is 0.01%^12^. Assuming 5% of mTBI children and 0.01% of the control children will report PPCS in the questionnaires, a power of 90%, and a 5% two-sided level of significance, 153 participants would be required in each arm.

Data were statistically analyzed using the MATLAB Statistics Toolbox, R2020b (MathWorks, Natick, MA).

## Results

### Study population

A total of 440 children who visited the ED between 2015 and 2020 due to brain injury, met the inclusion criteria for the mTBI group. From them, 142 children were excluded due to their past medical history, or the severity of the TBI as detailed in the exclusion criteria, 74 children could not be reached, and 19 refused to participate. Accordingly, 205 patients gave their consent, completed the RPQ questionnaire and were included in the final analysis.

**Figure 1:**
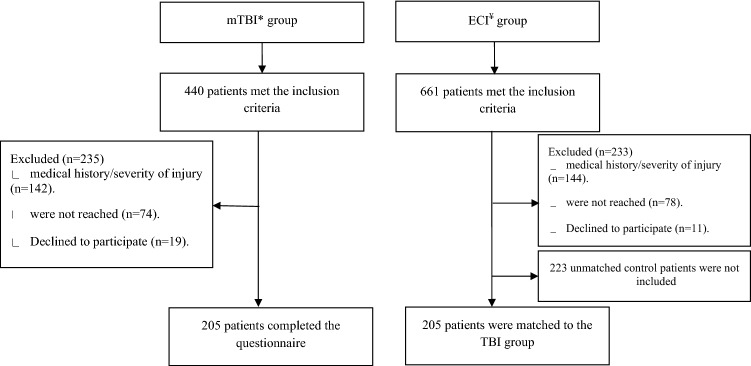
Flowchart of the patients in the study. *mTBI-mild traumatic brain injury. ^¥^ECI- extracranial injury.

In these same years, a total of 661 children were admitted to the ED and met the inclusion criteria for enrollment to the ECI group. From them, 144 children were excluded due to their medical history, because they suffered from multi-trauma or because they had a severe radius fracture that necessitated reduction. Accordingly, 205 children were matched to the mTBI group. The patients’ flowchart is detailed in Fig. 1.

The characteristics of the mTBI group and the control group are summarized in Table 1. For unmatched variables, there was not significant difference between the groups except for the mechanism of injury. Fall injuries were less common in the mTBI group (51 (24.9%) vs. 122 (59.5%), *p* < 0.0001), and none of the control group was injured in a motor vehicle accident.

**Table 1:** Baseline characteristics.

	mTBI group	Control group	*p*-value
N	205	205	
Age (years)	10.97 ± 2.16	10.89 ± 1.90	0.716*
Adolescents (age ≥ 13 years)	49 (23.9)	44 (21.5)	0.637
Sex (males)	138 (67.3)	140 (68.3)	0.916*
Time from injury (months)	33.50 ± 15.22	33.80 ± 15.24	0.844*
**Mechanism of injury**			
Fall	51 (24.9)	122 (59.5)	< 0.001
Sports related	72 (35.1)	79 (38.5)	0.539
Motor vehicle accident	44 (21.5)	0 (0.0)	
Other	38 (18.5)	4 (2.0)	< 0.001

Data presented as n (%); continuous data, mean ± SD.

*MP match parameter.

### Epidemiology and symptoms of persistent PCS

The prevalence of PPCS was significantly higher in the mTBI group as compared to PPCS like symptoms the ECI group (*p* < 0.001). In the mTBI group, a total of 52 patients (25.3%) had PPCS with a mean RPQ score of 3.43 ± 6.67 (range, 0–48). In the control group, there were only five patients (2.4%) with PPCS like symptoms with a mean RPQ score of 0.69 ± 2.25 (range, 0–24).

**Figure 2:**
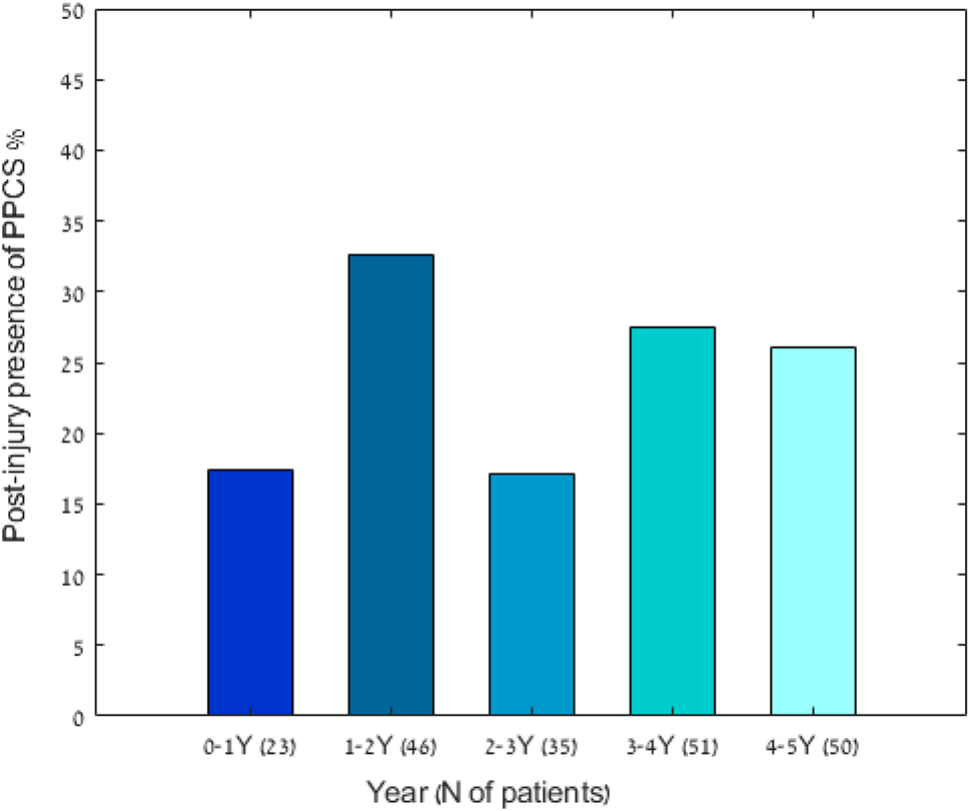
Presence of persistent post-concussion syndrome, 1–5 years after mild traumatic brain injury. Presence of persistent post-concussion was diagnosed based on the Rivermead Post-Concussion Questionnaire (*p* = 0.489).

**Figure 3:**
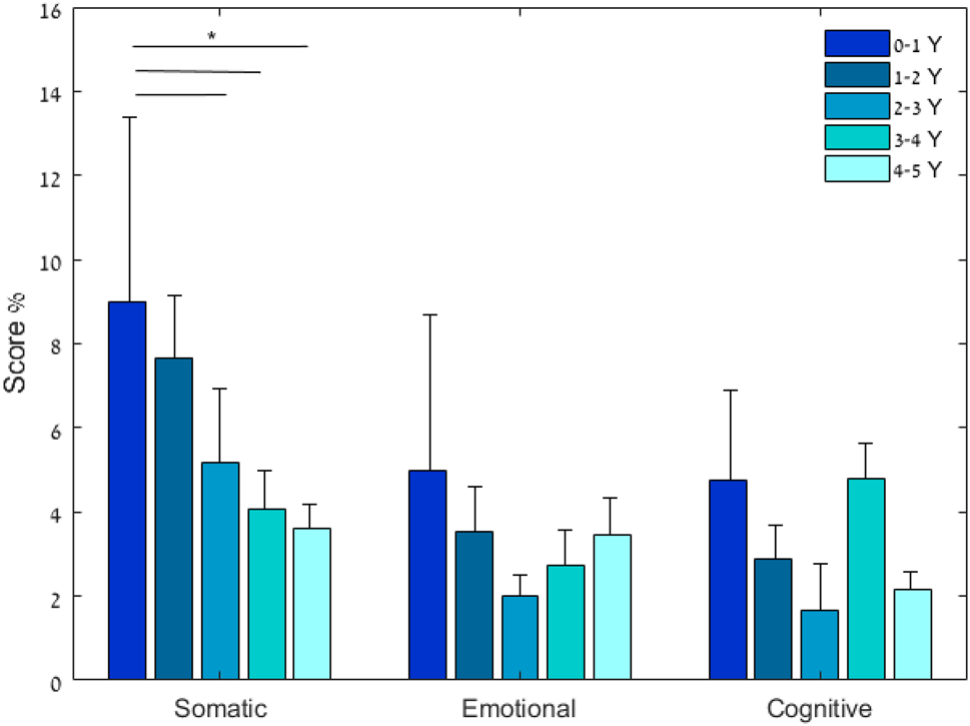
Rivermead Post-Concussion Questionnaire symptom severity scores presented as a three-model symptom factors (somatic, emotional, cognitive) in symptomatic post-mild traumatic brain injury children (N = 52). Rivermead Post-Concussion Questionnaire (RPQ). Values are presented as percentage of severity level: Somatic [0–36], emotional [0–16], cognitive [0–12]^21^. The somatic symptoms score severity was significantly lower 24 months after injury (*p* = 0.017).

The post-injury presence of PPCS at 0.5–5 years is illustrated in Fig. 2. The prevalence of PPCS, in the range of 0.5–5 years after the concussion, was not influenced by the time that elapsed since the injury (*p* = 0.489). However, among the mTBI symptomatic patients, after 2 years, the prevalence of somatic symptoms was significantly lower (*p* = 0.017), while cognitive and emotional symptoms were similar (Fig. 3). The mean RPQ score in the subgroup evaluated 2 years after the concussion was 11.94 ± 8.56 (range, 6–48).

**Figure 4:**
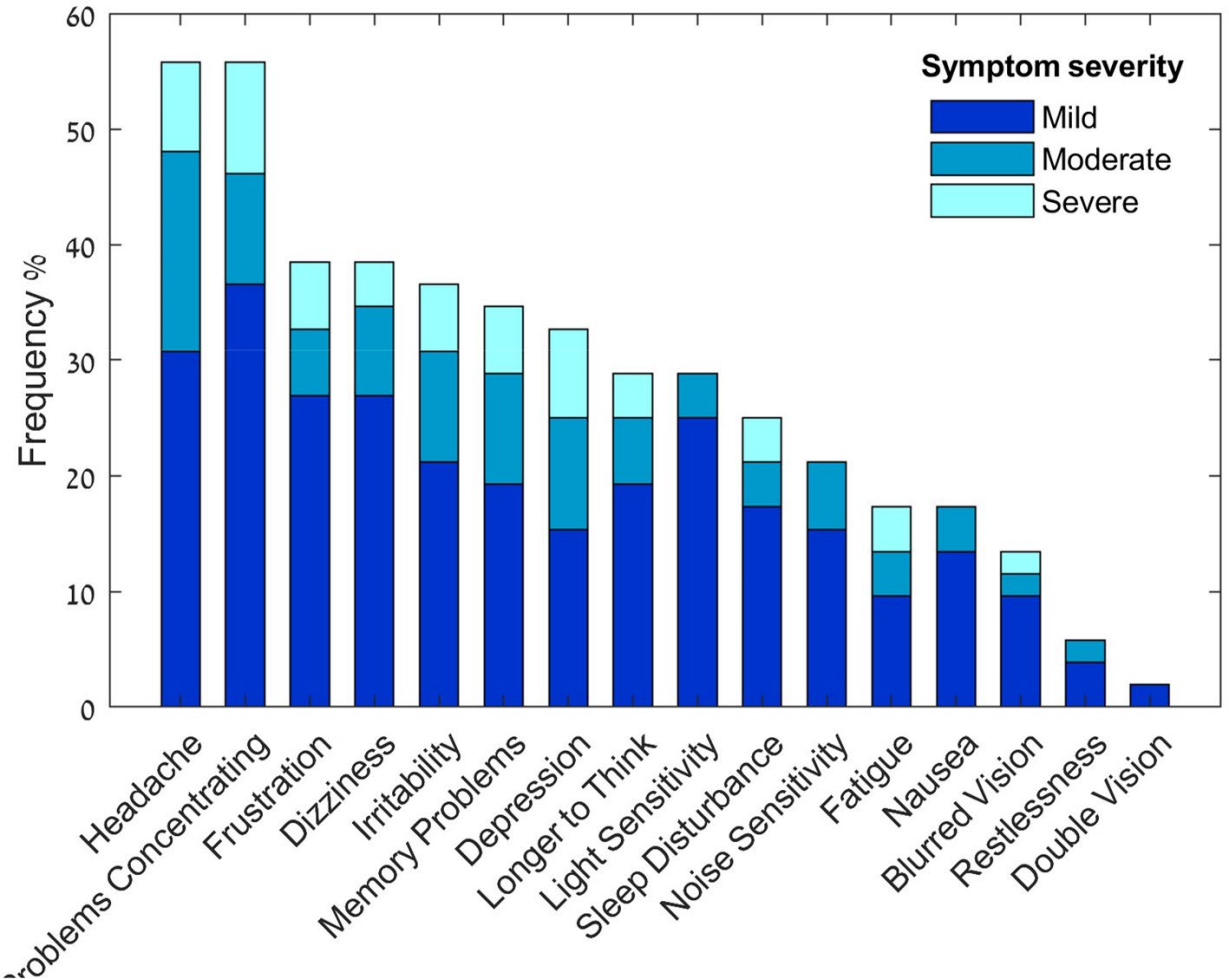
Frequency and severity of symptoms for children with persistent post-concussion syndrome after mild traumatic brain injury (n = 52).

The most common symptoms among children with PPCS were found to be headaches and difficulty concentrating. Sixty five percent of the symptoms were reported as mild, and only 13.2% were reported as severe. The frequency and severity of each of the PPCS related symptom are detailed in Fig. 4.

After evaluating the electronic medical records of these patients, we found that none of the patients who were found to have PPCS were categorized/labeled as suffering from persistent post-concussion syndrome.

### Prognosticators of PPCS

**Figure 5:**
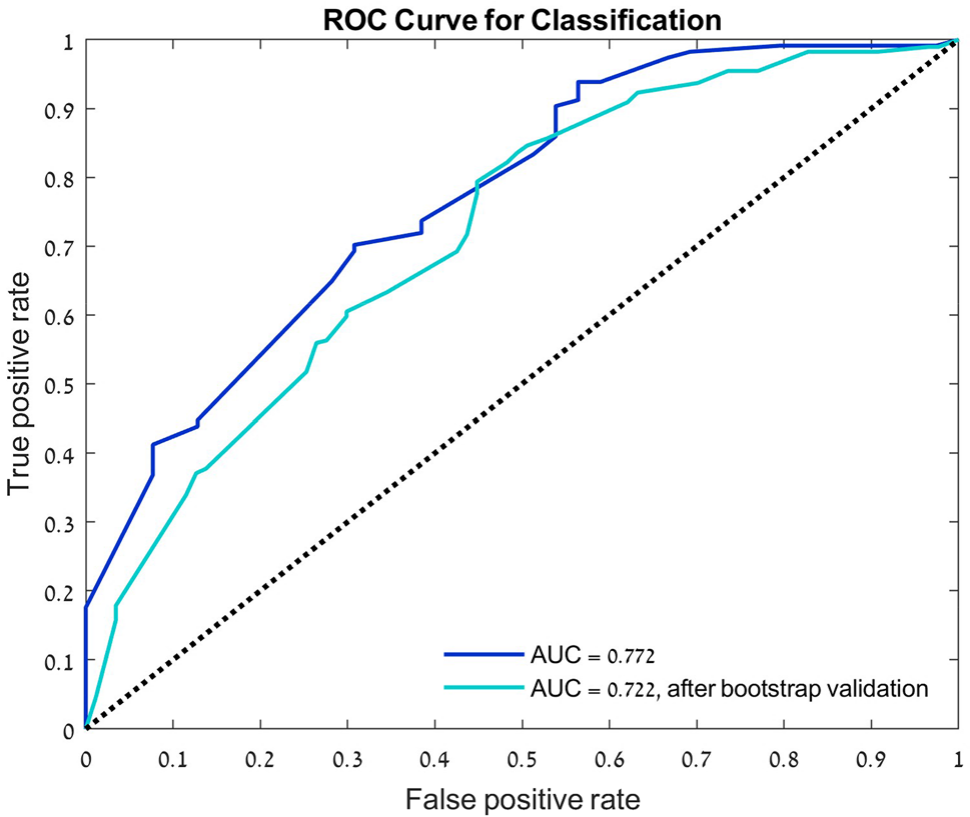
Validity of the predictive performance. Receiver operating characteristic (ROC) curves for the classification the multivariate logistic regression model.

A multiple logistic regression model was performed to evaluate significant predictors for PPCS in the mTBI group. A total of 153 (74.6%) participants had a complete data set of predictor variables for being included in the prediction model. Brain imaging data was missing in 25.4% of the patients. Univariate logistic regression analysis was performed for 13 predetermined variables as listed in Table 2. Five variables (gender, adolescents, vehicle accident, LOC, and imaging finding) were found to be covariates (*p* < 0.2) and accordingly, were evaluated in a multivariate model. The odds ratios of the model predictors are shown in Table 2. Significant predictors for PPCS were trauma caused by motor vehicle accidents (OR, 5.69 [CI 95%, 2.26, 14.3], *p* < 0.001), and age ≥ 13 years (OR, 2.93 [CI 95%, 1.12, 7.68], *p* = 0.029). Females more commonly suffered from PPCS as found in the univariate analysis (OR, 1.96 [CI 95%, 1.01, 3.76], *p* = 0.042), but it did not reach statistical significance in the multivariate analysis (OR 2.21 [CI 95%, 0.95, 5.10], *p* = 0.064). The multivariate logistic regression model demonstrates fair ability to predict PCS with an area under the curve (AUC) of 0.772, which decreased to 0.722 after bootstrap validation (Fig. 5).

**Table 2:** Logistic regression model for PPCS.

Variable	OR	CI 95%	*p*-value
**Univariate analysis**			
Female	1.96	[1.01, 3.76]	0.042
Age*	0.97	[0.84, 1.13]	0.722
Adolescents (≥ 13)*	1.98	[0.93, 4.20]	0.075
Time after injury	1.00	[0.98, 1.02]	0.672
Vehicle accident	4.99	[2.44, 10.19]	< 0.001
Loss of consciousness	0.47	[0.20, 1.07]	0.071
Amnesia	0.70	[0.34, 1.45]	0.335
Dizziness/ Fatigue	0.76	[0.39, 1.46]	0.404
Vomiting	0.69	[0.35, 1.35]	0.277
Headache	1.15	[0.62, 2.17]	0.654
Head injury signs	0.87	[0.45, 1.68]	0.674
Imaging findings^¥^	0.50	[0.21, 1.14]	0.099
Hospitalization	1.39	[0.67, 2.98]	0.380
**Multivariate analysis**			
Females	2.21	[0.95, 5.10]	0.064
Adolescents (≥ 13)*	2.93	[1.12, 7.68]	0.029
Vehicle accident	5.69	[2.26, 14.30]	< 0.001
Loss of consciousness	0.28	[0.10, 0.81]	0.019
Imaging findings^a^	0.46	[0.18, 1.16]	0.101

*At time of injury; *OR* odds ratio, *CI* confidence interval.

^a^Extracranial soft tissue traumatic finding or cranial fracture alone.

## Discussion

In this study, the long-term prevalence of persistent PCS symptoms was evaluated in 205 children after mTBI and was compared to children after ECI, 6–60 months after the injury. At this time frame, 25.3% of children after mTBI suffered from PPCS as compared to only 2.4% in the ECI group that showed PPCS like symptoms. In addition, there was no significant change in the prevalence of PPCS in correlation to time elapsed from the injury.

The high rate of PPCS after mTBI found in this study population, 25.3%, is similar to the findings of a recent study done by Ewing-Cobbs et al. In their study, 25% had PPCS and 39% continued to act differently one year after mTBI^13^. The prevalence of PPCS found in our pediatric population is in step with the adult population after mTBI, in which PPCS prevalence one year after the injury is reported to range from 5 to 30%^24–28^.

While comparing the prevalence of PPCS 6–60 months after the acute injury, there was no decline in the prevalence of PPCS. This could suggest that those who have continuous symptoms of PPCS more than six months after the acute injury are expected to have a chronic unremitting syndrome. This is in agreement with previous studies that found that patients suffering from PPCS are less likely to spontaneously recover^29–31^. The understanding that an evaluation at 6 months post-injury can predict the long-term consequence of the concussion may be helpful in creating a medical policies/algorithms in which anyone who comes to the ED because of TBI should be screened for PPCS 6 months after the injury.

To identify a selected subgroup that is more prone to develop PPCS and accordingly, needs more attention and a tighter follow-up policy, we conducted a multivariable analysis. We found that high velocity injury (MVA) and adolescence represent risk factors for PPCS. In addition, females were more prone to suffer from PPCS, but this was not statistically significant in the multivariant analysis. Our findings are in line with other studies^11,13,20^. Previous studies have theorized that the reason that adolescence and female are more prone to develop PPCS stems from the higher prevalence of mood disorders and anxiety seen in these subgroups^32–34^. In our study, patients with known psychiatric comorbidities were excluded, and still adolescence and females were found more likely to suffer from PPCS. However, we cannot rule out the possibility that these comorbidities were undiagnosed, as this study did not include a psychiatric evaluation^35,36^. Surprisingly, loss of consciousness was found to be related to lower rates of PPCS. However, it is important to note that loss of consciousness (LOC) was not seen by any medical staff but rather been reported by the patients. It might be possible that LOC reports were inaccurate and that can also explain the relative high rate of LOC (24.9%). None of the patients in this study were reported to be unconscious upon arrival or during their stay in the ED.

In 2018, the Centers for Disease Control and Prevention (CDC), published an mTBI management guideline for healthcare professionals, recommending clinical follow up and the use of validated symptom rating scales in children after mTBI^37^. Despite that, PPCS is still underdiagnosed in the pediatric population and the chronic unremitted symptoms are not being categorized as related to the mTBI^12,24–28,38,39^. In this study, none of the 52 patients who had PPCS were officially categorized as such in their electronic medical records by their pediatric primary care physician or neurologist. Needless to say, it is highly important to appropriately diagnose PPCS, since these children are prone to deficits in attention and cognitive control, school-related problems, inferior academic achievements, and truancy^20,40,41^. Furthermore, recent studies have shown that children with a delayed diagnosis of concussion are at a higher risk for persistent symptoms^42^.

The current study has several limitations. First, this study was not prospective from the injury time, and recall bias may have influenced this study. Second, since this study has relied on parental reporting, it is possible that they were not aware of all PCS symptoms afflicting their child. However, the same method of data collection was done in the control group, so the huge difference in the prevalence of PPCS symptoms between the groups, 25.3% vs 2.4%, indicates that the high prevalence of PPCS is likely to be true. Third limitation related to the study inclusion and exclusion criteria. Only children that were examined in the ED and underwent a brain CT scan and/or were hospitalized for at least one day of observation due to their injury were included. The decision to perform a CT or to hospitalize after a head injury is based on clinical judgment and may represent the more significant cases of mTBI. Since most cases of mTBI are not treated in the ED and not hospitalized^3,43^, this may lead to selection bias among the heterogonous group of mTBI. Forth, it is possible that parents to children suffering from PPCS were more likely to participate in the study and thus resulting in another selection bias. However, among the reached parents, only 7.3% declined to participate. Fifth, the retrospective nature of this study limited the access to the available clinical and psychosocial variables for analysis of prognosticators of PPCS. Last, there are some limitations regarding the diagnostic tool used in this study. Although the RPQ questionnaire has been widely used in the field of pediatric PCS, it has not been validated in this population^17–20^. In this study, a 3-factor structural model was employed to further analyze the RPQ that were PCS compatible. This model has been used in past research and its sub-division to symptom categories (cognitive, somatic, and emotional) mirrors the range of symptoms in the diagnostic criteria for PCS. It is worth mentioning that different studies have reported various possible factorial structures for the analysis of the RPQ, and to date, no preferred model has not been established^21,22,44,45^.

Despite these limitations, this multicenter study has several strengths, namely, high enrollment percentage of the eligible patients and the matching of a control group based on similar demographic characteristics.

## Conclusions

PPCS in the pediatric population is underdiagnosed. Twenty-five percent of the children admitted to the ED due mTBI may suffer from persistent symptoms years after the acute event. Unfortunately, in the vast majority, the diagnosis is missed and physicians, medical and teaching stuff, who are involved with these children should be aware of the cause and effect related to mTBI. These findings warrant better screening guidelines, and practices to be employed in the pediatric population after suffering mTBI. Once diagnosed, patients can be referred to appropriate medical, academic, and emotional consultations and interventions.
